# Conjunctival Capillary Hemangioma

**DOI:** 10.7759/cureus.1892

**Published:** 2017-11-29

**Authors:** Alanna Nattis, Henry D Perry, Eric D Rosenberg, Rubina Cocker

**Affiliations:** 1 Ophthalmology, Lindenhurst Eye Physicians and Surgeons, P.c.; 2 Ophthalmology, Nassau University Medical Center; 3 Ophthalmology, Westchester Medical Center; 4 Pathology, Hofstra Northwell School of Medicine

**Keywords:** conjunctiva, hemangioma, ocular surface, vascular tumors

## Abstract

Conjunctival hemangioma over the age of 60 is rare, with few cases reported in the literature. We present a unique case of a conjunctival capillary hemangioma, adding to the sparse literature of this uncommon vascular tumor. Here, we present an interesting case of spontaneous development of this tumor at age 68, without associated systemic disease process or cutaneous manifestations.

## Introduction

Vascular tumors of the conjunctiva are uncommon; capillary hemangiomas of the conjunctiva are quite rare [1-6]. Here, we discuss de novo development of a conjunctival hemangioma in a 68-year-old patient, as well as rapid documented growth--a presentation not previously noted in the literature [1-6]. 

## Case presentation

**Figure 1:**
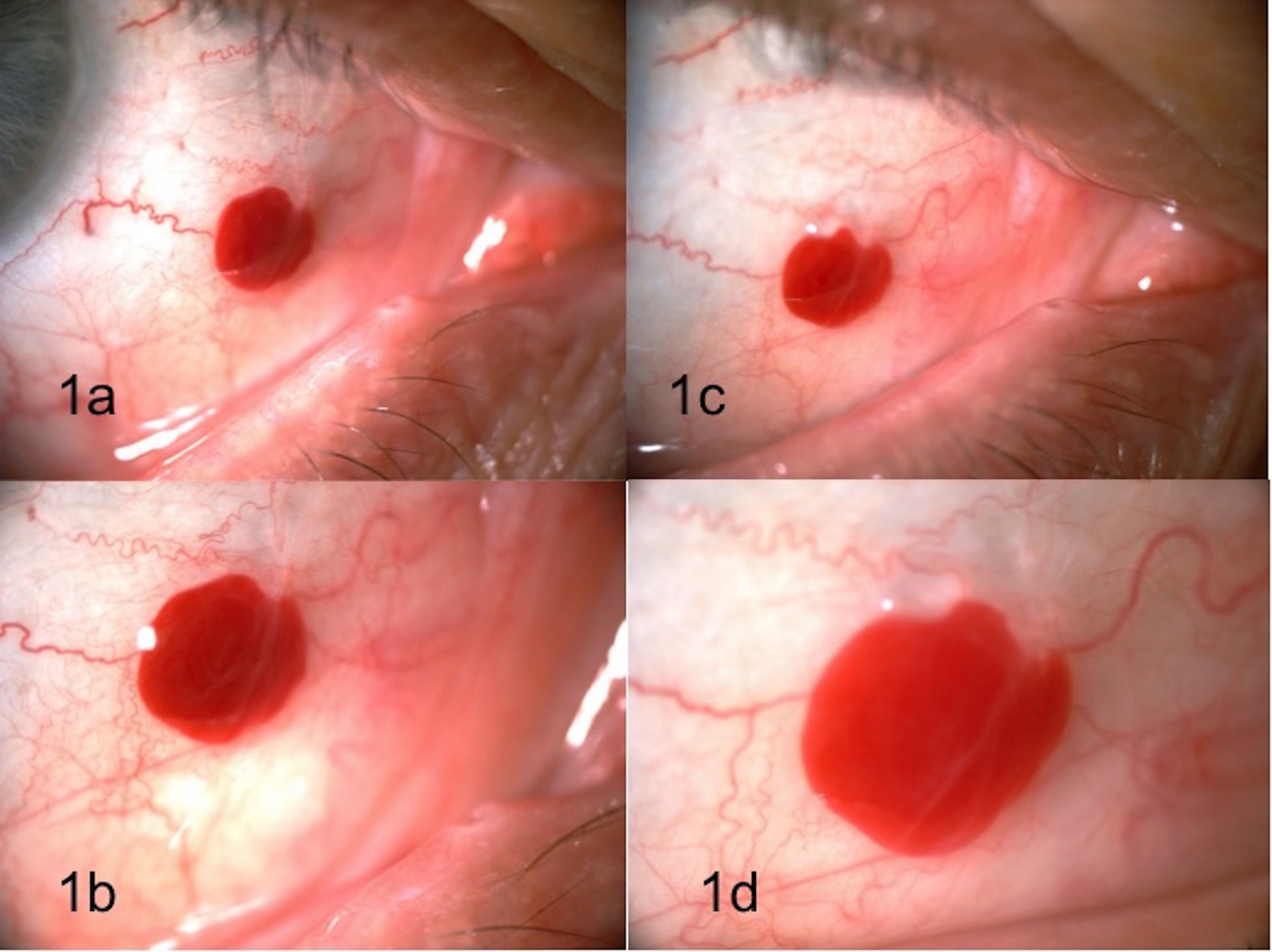
Conjunctival lesion (slit lamp photos) Conjunctival lesion at presentation (1a, 1b) and at six week follow up (1c, 1d). Images 1b and 1d are magnified photos of the lesion.

A 68-year-old male presented with a ‘blood filled cyst’ in his right eye of three months duration. The patient denied pain, change in vision, size, or color of the lesion over this time period. He denied eye trauma, allergy, or use of medications (including) blood thinners and non-steroidal anti-inflammatory drugs (NSAIDS). His past medical history was significant for trans-sphenoidal surgery for pituitary macroadenoma two years prior. His best-corrected visual acuity was 20/25 in both eyes. The ophthalmic evaluation including a dilated fundus examination was unremarkable except for the slit lamp exam of the right eye which revealed a 2.5 mm, partially mobile, solid conjunctival hemorrhagic lesion with well-defined borders, without feeder vessels or overlying lissamine green stain. The tumor is demonstrated in Figures 1a-1b.

The clinical diagnosis at that time was conjunctival varix or conjunctival hemangioma. We suggested observation as the lesion did not appear suspicious of malignancy (or growth per the patient), and followed up six weeks later.

On return, the lesion appeared to have grown in size by approximately 0.5 mm. This is demonstrated in Figures 1c-1d.

The lesion was excised completely and sent for histopathologic analysis. The patient was treated with a course of topical antibiotic and steroids and healed well.

**Figure 2:**
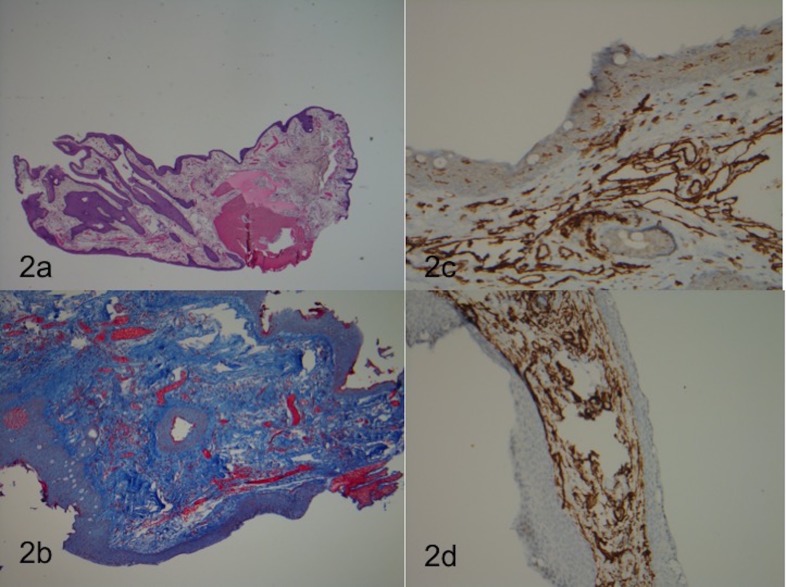
Pathology of conjunctival lesion Histologic evaluation of resected conjunctival tumor. 2a: H+E stain; 2b: Masson Trichome stain; 2c: CD31 stain, 2d: CD34 stain

The conjunctival biopsy showed subepithelial proliferation of variably thin-walled vascular channels, lined by a single layer of endothelial cells; some filled with blood. Extravasated red blood cells were also identified; trichrome stain revealed prominent stromal collagen and minimal fibrosis within the walls of only a few of the lesional vessels. The lesion was negative for desmin stain (no muscle differentiation in the vessel walls), and positive for CD31 and CD34 stains, confirming the presence of vascular endothelial elements. The final pathologic diagnosis was conjunctival capillary hemangioma. Photos of the histology slides are shown in Figures 2a-2d.

## Discussion

Conjunctival capillary hemangioma is a rapidly developing tumor derived from endothelial cells that has rarely been reported in patients over 60 years old [3, 6].  Most cases of reported conjunctival hemangiomas are congenital, asymptomatic, and said to involute by age seven [6]. These tumors may be isolated or associated with other ocular capillary hemangiomas (e.g. Sturge Weber Syndrome) [1].

Pathology of this lesion usually shows an intact surface epithelium with positive markers for vascular endothelium and pericytes (e.g. CD31, CD34, IA-4)—as did ours [1, 3]. 

Management of these lesions depends on the presumptive diagnosis, size, and extent of the lesion [2, 6]. Serial observation, incisional/excisional biopsy, cryotherapy, chemo/radiotherapy, modified enucleation, exenteration are all possible options, depending on suspicion, growth, and nature of the tumor [2, 6]. In addition, there have been isolated case reports of resolution of these lesions with timolol eye drops, similar to the treatment of cutaneous capillary hemangiomas in infants with propranolol [5]. However, most patients do well with excisional biopsy (if needed) and do not require further therapy [2, 6].

## Conclusions

As with all tumors of the conjunctiva, it is important to manage each case individually and observe for unusual characteristics and growth pattern. Our case is unique from those previously published in the literature, in that our patient spontaneously developed this tumor at age 68 without any associated systemic disease process or cutaneous manifestations. Most importantly, relatively rapid growth was documented photographically.
